# Effect of the Introduction of Reactive Fillers and Metakaolin in Waste Clay-Based Materials for Geopolymerization Processes

**DOI:** 10.3390/molecules26051325

**Published:** 2021-03-02

**Authors:** Caterina Sgarlata, Alessandra Formia, Francesco Ferrari, Cristina Leonelli

**Affiliations:** 1 Department of Engineering “Enzo Ferrari “, University of Modena and Reggio Emilia, 41125 Modena, Italy; cristina.leonelli@unimore.it; 2 Sibelco Ankerpoort NV, 6223 EP Maastricht, The Netherlands; alessandra.formia@sibelco.com; 3 Sibelco Italia S.p.A., 41053 Maranello (MO), Italy; francesco.ferrari@sibelco.com

**Keywords:** calcined clay, waste clayey, chemical stability, waste glass, metakaolin

## Abstract

In this study, the role of two reactive fillers, specifically a sand from a clay washing process as an alternative to waste glass powder and a commercial metakaolin (MK), into the geopolymerization process of waste clay-based materials was assessed. Three kinds of clayey wastes from mining operations—halloysitic, kaolinitic and smectitic clays—were tested as potential precursor of geopolymeric materials in view of a potential valorisation of these by-products. A mix-design based on the addition of low percentages (20%) of these fillers or MK to improve the mechanical and chemico-physical properties of geopolymeric formulations was evaluated. All the clays were thermally treated at a temperature of 650 °C, while the geopolymeric pastes were cured at room temperature. In particular, the chemical stability in water (pH and ionic conductivity of leachate water, weight loss), the variations in the microstructure (XRD, SEM), and in the mechanical performance (compressive strength) were analysed. The most reactive additive was MK, followed by sand and waste glass at very similar levels—1:1 or 2:1—depending upon the type of the clay but not strictly related to the clay type. The increase of geopolymeric gel densification due to the presence of MK and sand was replaced by a crack deflection mechanism in the case of the WG grains. The worst performance (chemical stability and mechanical properties) was found for the halloysitic clay, while kaolinitic and smectitic clays developed strengths slightly below 30 MPa.

## 1. Introduction

Clays and clayey mineral have been used as building blocks since the dawn of humankind. Lately it seems there has been renewed interest in them due to their low environmental impact [1]. Technological knowledge has led to engineered clay-based materials that show high performance without firing but rather adopting a cold sintering/consolidation in highly alkaline or acidic media. Such a process, known as geopolymerization [2] consolidates aluminosilicate minerals—mainly clays—and different types of inorganic wastes containing high percentages of silica and alumina, i.e., slag, fly ash, volcanic ash, etc.

Clays and kaolinite have been widely used as starting materials to synthesize geopolymers. Generally, to enhance the reactivity of the clay in alkaline media kaolinite is calcined at 600–800 °C to produce metakaolin, a well-known artificial pozzolanic material. The dehydroxylated kaolin product has a higher amount of Al(IV) and Al(V) that allows the formation of well cross-linked geopolymeric matrices [3].

Halloysite and kaolinite have an identical chemical composition, except for the fact that halloysite may have as many as two molecules of H_2_O as interlayer water. The additional water in the interlayers of halloysite is a decisive influence upon its crystal morphology, which is generally a curled rather than a plate-like form as occurs in kaolinite. Common forms are elongated tubes and spheroids [4] which demonstrate that the thermal transformation of halloysite is largely similar to that of kaolinite, providing a meta-halloysite form that has already been tested in geopolymer formation [5,6]. Halloysite-rich kaolin was also successfully used for the realization of geopolymers by Tironi et al. [7], Zhang et al. [8] and Palmero et al. [9]. To overcome the high cost and chemical aggression associated with classical alkaline solutions, experimental investigation was conducted on the alkali activation of a kaolinitic clay using an alkaline mixture composed of hydrated lime (Ca(OH)_2_) and sodium carbonate (Na_2_CO_3_) solution [10]. Durability tests on samples soaked in water for 24 h showed a reduction in strength from 34 to 22 MPa for specimens prepared with NaOH solution, and from 21 to 11 MPa for the specimens prepared with a Ca(OH)_2_/Na_2_CO_3_ alkaline mixture. For specimens produced in this work it was decided to use the classical sodium hydroxide (NaOH) activator with sodium silicate (Na_2_SiO_3_) as alkaline solution, considering that the recorded strength value of specimens produced using a Ca(OH)_2_/Na_2_CO_3_ mixture as the alkaline activator was 35% less than that achieved by the classical NaOH solution.

It has already been demonstrated that a one-size-fits-all approach for processing and activating clay minerals is not viable [1]. Instead, activation routes need to be tailored according to the clay mineralogy to achieve the binder properties required for key applications. In particular, clay resources offer many opportunities for use in alkali-activated materials (AAMs), but many challenges remain. There is a spectrum of reactivity between different clay minerals in their natural state, likely determined by a combination of layer structure, morphology and degree of amorphousness [11]. The reactivity can be enhanced through calcination treatments [5,6], but the 3D reticulation of the network of aluminosilicate formed is primarily determined by the molar ratio of soluble Si:Al in the system. Geopolymers are favored with respect to crystalline zeolite when Si:Al > 1.5. This limit has been empirically established [1,12] yet addition of SiO_2_ [13] or SiO_2_ and Al_2_O_3_-rich fillers/additives [14,15] helps to increase such Si:Al ratio.

**Figure 1:**
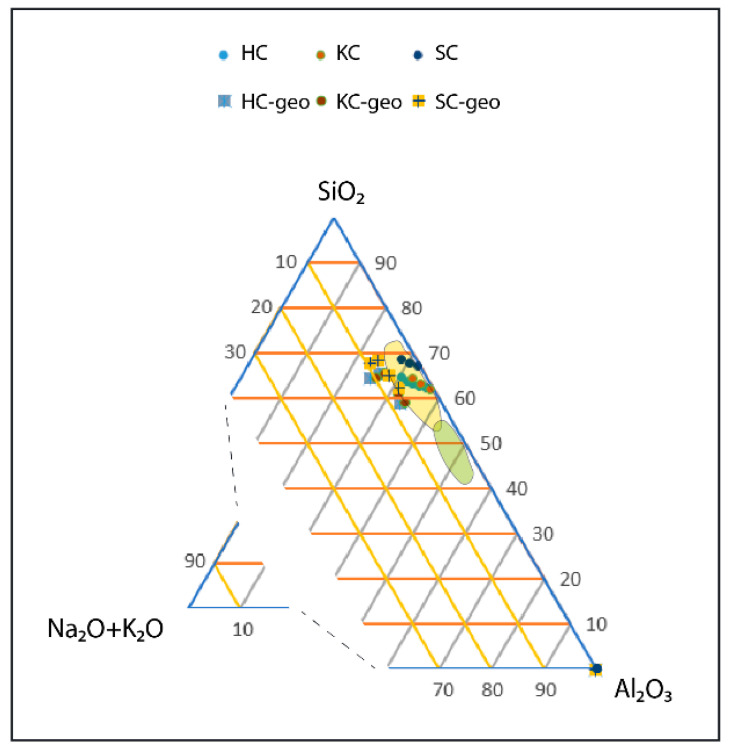
Ternary diagram reporting the as received raw materials and their corresponding geopolymers with respect the two separate areas of typical 2:1 clay minerals (yellow area, richer in SiO_2_) and 1:1 clay mineral (green area, richer in Al_2_O_3_). Data for the two phyllosilicate areas have been elaborated from [1].

In this work we investigated the consolidation via alkali activation of three types of clay minerals, belonging to the kaolin 1:1 and illite 2:1 clay group (Figure 1). The aim was to compare the chemical and physical properties of geopolymers made with these clays after calcination, and to see how the different structures of the clays influence the properties of the final materials alone (NF) and when mixed with sand (SA), waste glass (WG) and metakaolin (MK). Concerning the chemical reactivity of these additions we know that calcined kaolinite, or metakaolin, has been well studied in recent years and has proven to have very good pozzolanic properties [16]. Waste glass from containers, when used in the form of very fine powders has also proved to be reactive in alkaline environments [13,17,18]. Sand is composed mainly of quartz, known to be extremely stable in the alkaline environments.

Therefore we expect a different degree of reactivity among the three additives, with being metakaolin the more reactive, followed by fine waste glass powders and finally by the quartz sand. These additives were added to the calcined clays and activated with a Na- based alkaline solution at room temperature to avoid the formation of zeolitic materials [19] but to develop a 3D aluminosilicate network, suitable for structural materials. In order to evaluate the chemical stability, we tested the consolidated geopolymers in water. We then measured pH and ionic conductivity of the leachate. We compared this information with the XRD mineralogical phases. The mechanical performance (compressive strength) was described on the basis of the microstructure observation and microfracture propagation (SEM-EDX).

The compositions analyzed in this work with KC (kaolinitic clay), SC (illitic/smectitic clay) and HC (halloysitic clay) are represented in the ternary diagram in Figure 1, as a function of the oxide percentage of SiO_2_, Al_2_O_3_ and Na_2_O + K_2_O.

## 2. Results and Discussion

Formulations of geopolymers prepared in this study are listed in Figure 1 and Table 1 with the respective percentages of additive added and the alkaline activators used. We optimized the amounts of NaOH and Na-silicate in a previous study [20] and we optimized the solid to liquid ratio (S/L) in this study in order to ensure better mould filling conditions. As it can be deduced by the S/L values reported in Table 1, HC required less liquid than the other clayey wastes. The amount of alkaline activators used for formulations is expressed by the corresponding NaOH to Na_2_SiO_3_ ratio.

From a visual inspection it was possible to assess that all the formulations after the extraction from the mould do not present the efflorescence typical of unreacted sodium solutions. Samples are smooth and finger pressure resistant, and especially with the addition of fillers or MK, all the samples appear very hard. These first observations, in particular the absence of efflorescence, are a good indication that the major part of the alkaline solution has reacted with the calcined clays as well as with the added fillers and MK.

### 2.1. Integrity Test and Weight Loss

From the integrity test results it was possible to attribute a positive qualitative value to the chemical stability of the samples. After 24 h in water the samples resisted without losing structural consistence. The liquid solution showed no color change, no sediment formation, and no change in consistency (see Supplementary Materials Figures S1 and S2).

**Figure 2:**
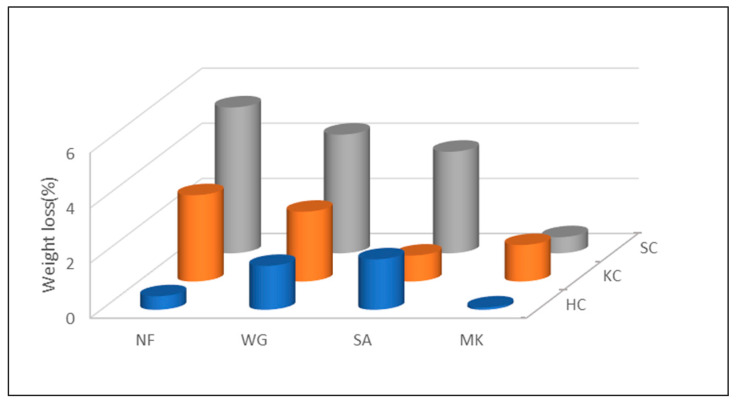
Comparison of weight loss results (%) of HC-, KC- and SC-based samples without (NF) and with the addition of fillers (WG, SAND, MK) after immersion in water for 24 h.

Weight loss after immersion in water increases when the reticulation of the geopolymer is poor (results are shown in Figure 2). Concerning the samples based on SC and KC clays, it can be noted that the weight loss values decrease with the addition of waste glass, sand and metakaolin, indicating a good reactivity of these additives in the alkaline environment of this formulation. This trend is different for samples made with HC clay, for which the addition of WG and sand seems to be deleterious. In addition, comparing the values of samples with and without fillers of the three clay typologies, the samples with the highest weight loss values are recorded for SC, followed by KC and then HC. For the series based on SC, it can be observed how the addition of waste glass reduced the weight loss, followed by the addition of sand, which proved to be very reactive in this formulation, then MK that gave the lowest weight loss value, being the more efficient reticulating additive. Addition of MK to the KC formulation does not have the same reticulation efficacy, indicating that the alkaline solution has already been consumed by the KC component.

### 2.2. pH and Ionic Conductivity

The reticulation reaction in geopolymer gel formation is often indicated as a geopoly-merization reaction [3]. This is a typical condensation reaction between two monomeric species, Si(OH)_4_ and Al(OH)_4_^–^ which are the result of the aggressive alkaline attack on amorphous aluminosilicate powders. When the alkaline solution is not completely reacted with the surface of aluminosilicate powders it still may be leached out during immersion in water of the densified geopolymeric gel. Hence, pH measurements can be an indirect method to evaluate the efficacy of reticulation reactions.

Results of pH measurements (see Supplementary Materials Figure S3) on the liquid solution recovered after the integrity test, show a stable trend of pH within an alkaline range (15–17) during the 24 h of the test, for all samples. There are no evident differences between samples with and without fillers. Therefore, a good chemical stability of the materials in alkaline media is confirmed as it appears for a reference material, a MK-based geopolymer, showing a pH value around 11 ± 0.2 [21,22].

Since pH is not sensitive enough to discriminate the differences in the 3D aluminosilicate network stability in water, we evaluated the ionic conductivity of the liquid obtained after the leaching test. The ionic conductivity of such a liquid is the result of the number and quality of ions released by the geopolymer network. As it will be explained in Section 2.3, these ions can be directly related to the number al Al^3+^ ions that are present in the tetrahedral coordination structure.

**Figure 3:**
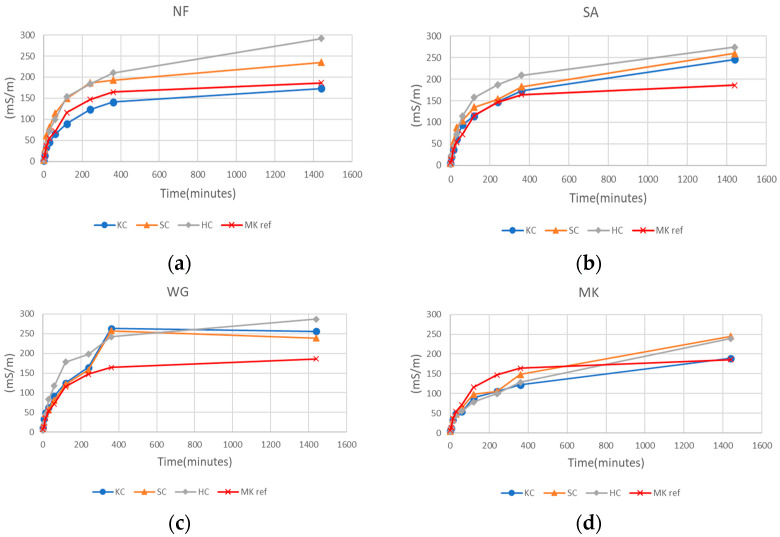
Ionic conductivity results of samples made with 100% clay (**a**), samples with clay and addition of sand (**b**), clay and waste glass (**c**), and clay with addition of metakaolin (**d**).

Ionic conductivity values follow the typical trend for alkali activated materials [21,23]: an increase in ionic conductivity with time, due to the release of ions into the water, especially during the first 60 min of the test. In Figure 3 the results are grouped in graph (a) samples with no filler, in graph (b) samples with addition of sand, in graph (c) samples with addition of waste glass, and in graph (d) samples with insertion of metakaolin.

**Figure 4:**
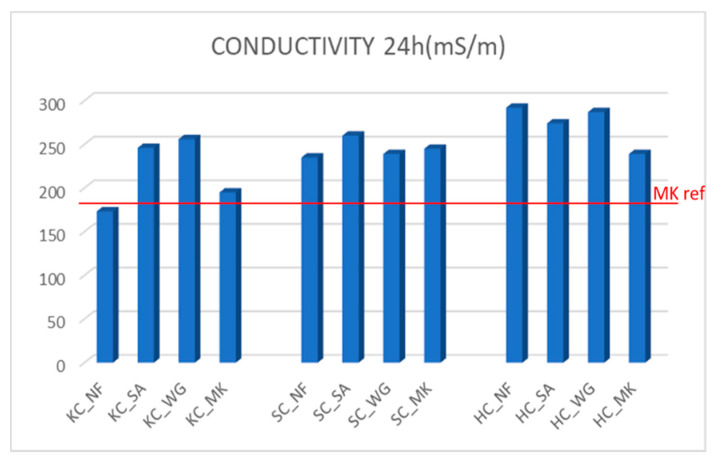
Ionic conductivity values of aal the samples after 24 h from test start. The line MK ref indicates the ionic conductivity of reference geopolymer based on 100% metakaolin.

It can be seen how the ionic conductivity tends to reach a stable value with time (Figure 3). In Figure 4 the last conductivity value measured during the test after 24 h for all the samples is reported. Samples with KC showed an increase of ionic conductivity for specimens with added sand and waste glass. For the SC series, the values are similar with or without inclusion of fillers. Samples with HC showed higher ionic conductivity, with a slight decrease for samples with fillers, in particular for HC_MK. The straight line reported in the plot and indicated as “MK ref” represents the value of the reference geopolymer based on 100% metakaolin, as an example of good geopolymerization/reticulation. Most of the samples show a degree of geopolymerization inferior to the MK-based geopolymer, especially when fillers are added. Nevertheless, the variance is not that high, and it is still within an acceptable interval, when compared to literature data [22,23,24].

### 2.3. Compressive Strength

**Figure 5:**
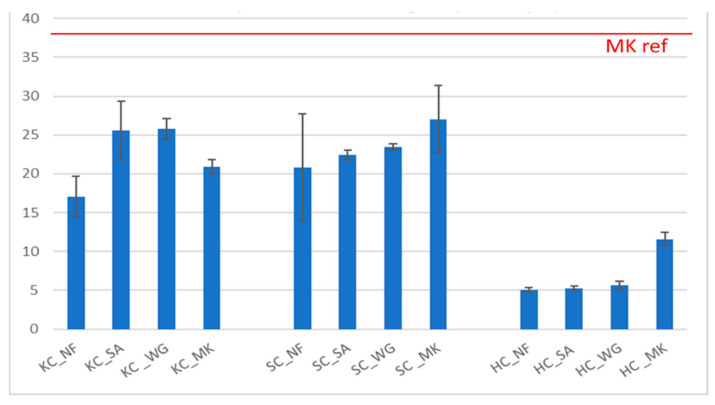
Compressive strength results of the geopolymers aged 28 days. The line MK ref indicates the compressive strength of reference geopolymer based on 100% metakaolin.

Mechanical properties were tested with compressive tests. Compressive strength increases with the introduction of fillers for KC and SC clay, in particular the values of KC_SA, KC_WG and SC_MK are between 25–30 MPa (Figure 5). The reference value of pure MK-based geopolymers is also reported [25]. Samples made with HC clay have very low compressive strength, also with introduction of metakaolin where it doubles the value, yet it still remains low. From this data, we can deduce that the smectitic clay can reach the highest strength value, since it retains a certain level of the 2:1 structure. When MK is added the strength increases, but this is not accompanied by an ameliorated chemical stability, as indicated above. These results may indicate a denser geopolyeric structure, with respect to other clays, which is not accompanied by the formation of strong covalent bonds. The lower alumina content, with respect to silica, typical of this aluminosilicate reduces the Na^+^ cations retained in the structure. In fact, it should be remembered that in the 3D geopolymer network the alternation of Al^3+^ cation and Si^4+^ cations is random. With the Al^3+^ cations in tethraedral coordination surrounded by four oxygens with 2− charges each, the electroneutrality is reached by bonding a Na^+^ ion with a strong ionic bond. In the smectitic clay, the presence of Al^3+^ cations is reduced, hence the capability to retain the monovalent cations of the alkaline activator is also reduced (see the ionic conductivity values in Figure 4).

### 2.4. X-ray Diffraction

**Figure 6:**
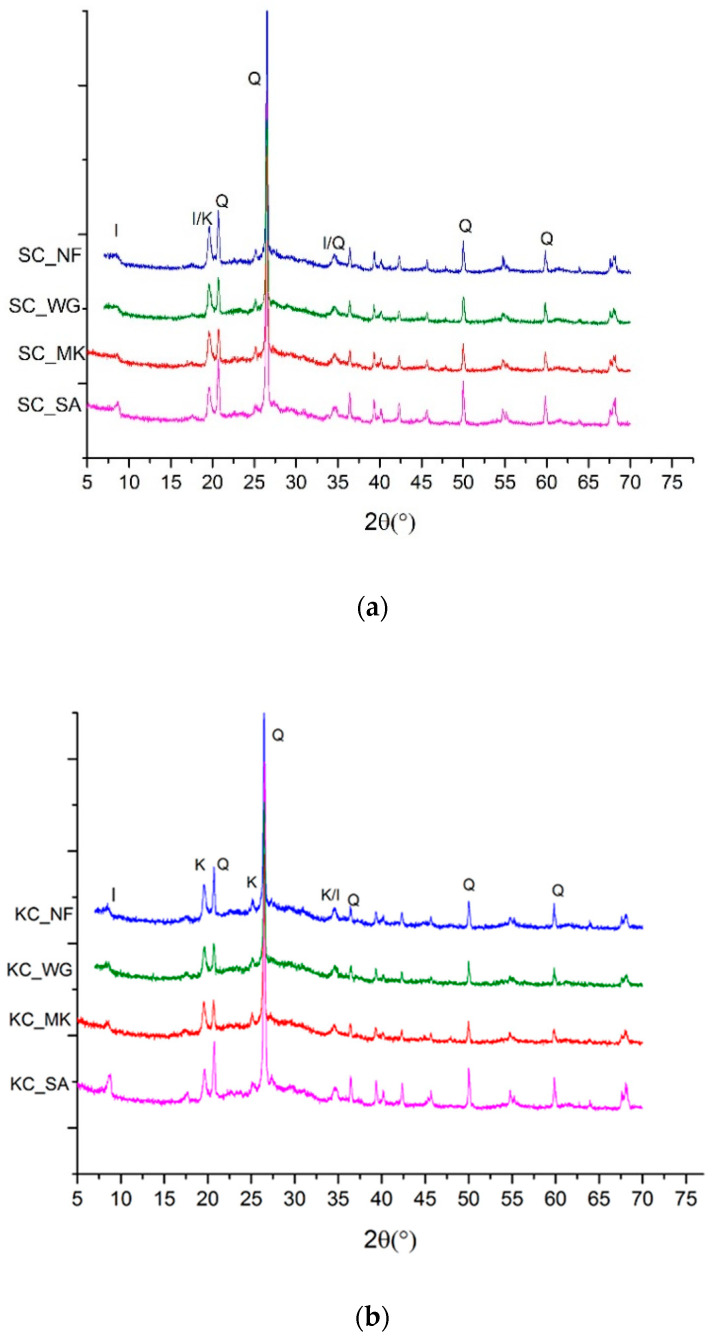

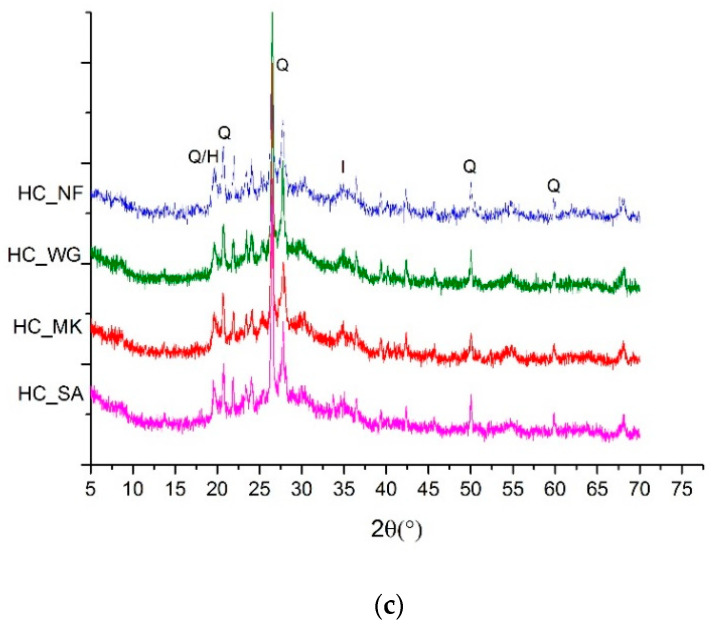
Comparison of X-ray diffraction spectra of clays and samples made with them: (**a**) samples with SC clay; (**b**) samples with KC clay; (**c**) samples with HC clay. (Q = quartz; I = illite; K= kaolinite; H = halloysite)**.**

In Figure 6 the X-ray patterns of samples made with calcined clays with and without additives are shown. Some typical peaks corresponding to kaolinite and halloysite with calcination of clays at 650 °C disappear, indicating a loss of crystallinity, as observed in the literature [16]. In fact, in X-ray diffraction lines of the samples, in particular for KC (b) and HC (c) clay, are not clearly visible. Comparing the XRD patterns of samples for each clay, there are no relevant differences depending on typology of the additive. Only for the addition of sand, it is possible to observe for all three spectra (a), (b) and (c) an increase of the peaks corresponding to quartz (Q) and illite (I).

Another interesting, as well as common, feature of the XRD patterns collected for these geopolymers is the presence of the broad band, typically a defined halo, characteristic of the amorphous phase. In metakaolin-based geopolymers such a halo is positioned, as in this case, between 25–32° in 2 theta. This halo is not shifted by the addition of additives, indicating that the overall signal of the amorphous phase, also indicated as geoplymeric gel, is retained. The chemical consequence of this structural characteristic has been discussed as a common pH value and a general homogeneity in the ionic conductivity of the leachate liquid after immersion.

### 2.5. Environmental Scanning Electron Microscope (ESEM)

**Figure 7:**
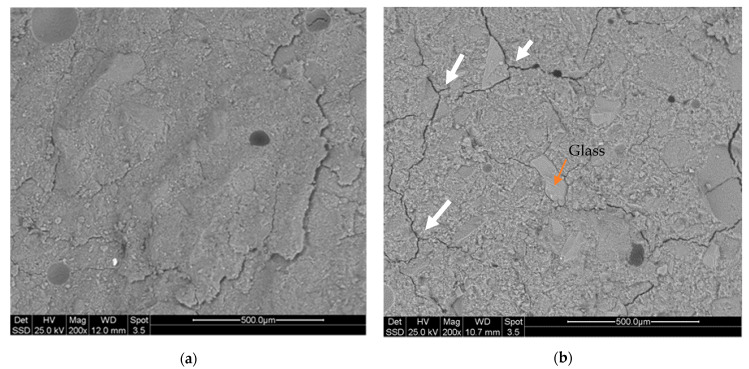
SEM micrograph of (**a**) KC_NF composition and (**b**) KC_WG composition after 28 days of curing.

For SEM micrographs, the compositions with the best mechanical performance and chemical stability were chosen. Microstructure images of KC_NF composition and KC_WG composition are shown in Figure 7. It is possible to observe for both samples a compact and homogeneous matrix after alkali activation and curing at room temperature for 28 days. In image (b) the grains of the added waste glass are distinctly visible, probably due to the high particle size of waste glass (as identified by EDS analysis). Comparing the two images, it is possible to obtain information about the different types of fracture paths of the samples and correlate them to the mechanical strength results. In fact, in KC_WG the fracture extends around the glass grains, acting as a reinforcement and delaying the breakage of the samples (see white arrows in Figure 7b). This could explain the increase of mechanical strength seen for KC clay with added fillers.

Using the x-ray fluorescence (EDS) device mounted on the SEM equipment, we obtained an overall semiquantitative analysis, which can be used to obtain an indicative value of Si/Al ratio and Na/Al to correlate with the occurring geopolymerization. The analysis in different points of samples confirms the chemical uniformity of the matrix and the Si/Al and Na/Al mass value for both samples of around 2 and 0.9, respectively (Table S1 in the Supplementary Materials).

The sand present acts as WG inclusions, increasing the crack deflection mechanism for the reinforcement of the overall structure, with the other samples showing the same microstructural behavior. The effect of MK addition can be explained by an increase in 3D reticulation of the geopolymer gel, as several studies have already highlighted. In the case of this additive, the Si/Al ratio is the factor that mainly affects the mechanical properties of the materials [26]. Crack deflection as a reinforcement mechanism was found also for SC-WG samples (Figure S5), while for the HC-NF sample the loose structure was efficiently densified only by MK addition (Figure S6). Additionally, the effect of very fine WG particles is to produce a denser matrix, as reported in the literature for fly-ash based mixes [27].

## 3. Materials and Methods

**Figure 8:**
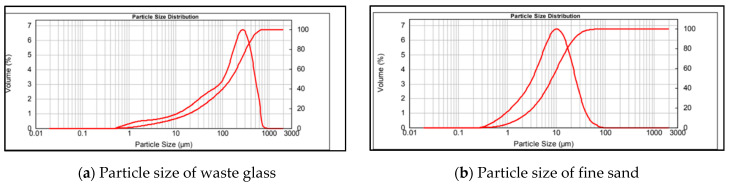
The particle size of waste glass (**a**) and fine sand (**b**).

Clays used as matrix in this research and the reactive fillers of waste glass and fine sand are supplied from Sibelco S.p.A.: HC (Fossanova, Italy), KC (Kingsteignoton, UK), SC (Donbass, Ukraine), SA (Robilante, Italy), WG (Cruy, France). All these materials are mining by-products obtained thanks to industrial waste recovery processes for reuse as “secondary raw materials”. In detail: clay KC, belonging to the kaolin 1:1 clay group and clay SC, belonging to the illite 2:1 clay group, come from the Donbass region in Ukraine, and Kingsteignton in the Southwest of England, respectively. Both these clays are presented in overlapping layers that are properly analysed and mixed to obtain a stable product over time in terms of chemical-technological performance; halloysitic clay HC belonging to the kaolin 1:1 clay group, is a co-product of the production of glass sands and comes from Lazio in the province of Latina in Italy. This clay is separated from the sand by washing and filtering. Waste glass (WG) used as filler in this study is a white cullet glass processed in Cruy (France), mainly characterized by oxides of 70%SiO_2_, 12%Na_2_O and 7%CaO. The particle size was reduced until dimension reported in Figure 7. The fine sand (SA) with 67% of quartz and 27% of mica/illite, is primarily characterized by oxides of 82%SiO_2_, 10%Al_2_O_3_ and 4%K_2_O and is a byproduct from quartz sand processing. The particle size is reported in Figure 8 compared to the waste glass particle size. 

The clays used for the preparation of samples were dried and sieved at 75 µm, and calcined at the temperature of 650 °C, accordingly to the thermograms (Figure S4 in the Supplementary Materials) and [28]. The mineralogical composition and the main oxides present in the clays are listed in Table 2. 

The white metakaolin (MK) added to the composition is a high purity pozzolanic additive for OPC (D90 10 µm) produced by Backstain (Köln, Germany), containing 52%SiO_2_ and 45%A_2_O_3_, and traces of α-quartz.

Sodium hydroxide solution (NaOH) 8M used as alkaline activator were prepared by dissolving NaOH pellets (Sigma-Aldrich Corporation, Burlington, MA, USA, purity ≥ 98%) in distilled water and stored to cool to room temperature. Sodium silicate solution (Na_2_SiO_3_) provided by Ingessil s.r.l. (Verona, Italy), with a molar ratio SiO_2_:Na_2_O = 3 was added to sodium hydroxide to complete the alkaline activator solution.

Four mixture groups/sets of samples are introduced in this research. The first series of samples (NF) was prepared with 100% of calcined clay without fillers. For the second series of samples (WG) 20% of waste glass was added to the clay, while for the third series (SA) 20% of sand was introduced, to evaluate the influence on geopolymerization process and the stability of the compositions. The last series of sample (MK) was prepared with clay and 15–20% of metakaolin. The activator solutions used in the formulation have a NaOH/Na_2_SiO_3_ ratio = 1. Samples were prepared mixing the powders with the activator solutions into a container with a mechanical mixer for approx. 10 min. When the geopolymeric paste was homogenous and achieved a good workability, it was poured into a mold covered with a plastic film. The samples were then cured at room temperature for 28 days.

The chemical stability was defined with an integrity test in water, weight loss and by pH and ionic conductivity measurement. The integrity test is a preliminary qualitative test to verify if the geopolymerization process has occurred. It consists of immersing a sample in distilled water with a solid/liquid ratio 1:100 for 24 h and evaluating the structural consistence [24]. The integrity test is associated with the quantitative measure of weight loss. The weight loss was evaluated, comparing the initial (w_i_) and the final (w_f_) weights expressed in percentage according to the following Equation (1):

Weight loss (%) = (w_i_ − w_f_)/w_i_ × 100 (1)

The sample was immersed for 2 h in acetone, dried in air and weighed (w_i_) and then dipped for 24 h in water. After 24 h it is put back in acetone for another 2 h, dried for a few hours and then weighed (w_f_) again.

To measure the pH and the ionic conductivity the sample was immersed in stirring condictions at 20 ± 2 °C in deionized water with a solid/liquid ratio of 1/10 for 24 h. Ionic conductivity and pH of the solution within the sample immersed were determined at different times (0, 5, 15, 30, 60, 120, 240, 360, 1440 min), to obtain a trend of the change in value during the 24 h and to obtain information on the amount of dissolved solid [21]. The pH was detected with a Hamilton type Liq- glass SL Laboratory pH sensor (Hamilton A.G., Bonaduz, Switzerland), and the electrical conductivity of the solution was measured with a calibrated cell both of which were connected to the digital display of pH 5/6 and Ion 6- Oakton/Eutech Instruments (Oakton Instruments, Vernon Hills, IL, USA) [29].

Cubic samples (2 × 2 × 2 cm^3^), after 28 days of curing, were tested to determinate compressive strength using a model 5567 Universal Testing Machine (Instron, Norwood, MA, USA) with 30 kN load limit and displacement of 1 mm/min according to the standard UNI EN 826 [30].

X-ray diffraction patters of clays and samples were recorded by a PW3710 diffractometer (Philips, Almelo, The Netherlands). Specimens were scanned from 5° to 70°, 2 theta range on powdered samples.

Morphology observations were conducted on fresh fractured samples by environmental scanning electron microscopy (ESEM) using a QUANTA 200 microscope equipped with EDS (FEI, Hillsboro, OR, USA).

## 4. Conclusions

Concerning the chemical stability of the geopolymers prepared starting from clay by-products, the results of integrity and weight loss tests have shown that samples made with the three types of clay have good stability and resistance in water. Weight loss values of less than 5% were confirmed by pH and ionic conductivity tests. As seen in Figure 3 and Figure 4, the conductivity increases when the ion release is higher; in fact, the higher the chemical stability of the sample is, the lower the ionic conductivity will be. Samples made with KC clay showed slightly lower conductivity values than the samples made with the other two clays, both with the presence or absence of fillers. Also, it can be noted that the addition of sand, waste glass and metakaolin does not always improve the chemical properties of samples, as seen in Figure 3. Specifically, the ionic conductivity of leachate water from HC_NF sample is 299 mS/m and decreases to about 275 mS/m for SA or WG addition decreasing further at 245 ms/m in the case of MK addition. On the contrary, KC-NF sample presents a leachate with ionic conductivity of 175 mS/m that rises to 250 mS/m with SA or with WG addition to decrease again towards 180 mS/m with MK. The SC series shows an intermediate situation, even though it belongs to a different type of clay, the 2:1 family. This observation indicates that a proper formulation and the most efficient additives should be sought for each single clay mineral in order to achieve either the best chemical stability or the best mechanical performance during the alkali activation.

Regardings mechanical properties shown in Figure 5, it was proven that the addition of fillers helps increasing the compressive strength for samples with KC and SC with a crack deflection mechanism typical of reinforcement particles. The improvements provided by the addition of MK are almost negligible for these two types of clay, while MK doubles the resistance of samples made with HC clay.

## Figures and Tables

**Table 1:** Formulations of samples made with HC (halloysitic clay), KC (kaolinitic clay), and SC (illitic/smectitic clay). S/L = solid to liquid ration, where solid is the clay + filler content.

Sample	Clay (%)	Sand (%)	Waste Glass (%)	Metakaolin (%)	NaOH/Na_2_SiO_3_	S/L
HC_NF	100	0	0	0	1	1.7
HC_WG	80	0	20	0	1	1.7
HC_SA	80	20	0	0	1	1.7
HC_MK	80	0	0	20	1	1.7
KC_NF	100	0	0	0	1	1.5
KC_WG	80	0	20	0	1	1.5
KC_SA	80	20	0	0	1	1.7
KC_MK	80	0	0	20	1	1.7
SC_NF	100	0	0	0	1	1.5
SC_WG	80	0	20	0	1	1.5
SC_SA	80	20	0	0	1	1.7
SC_MK	80	0	0	20	1	1.7

**Table 2:** Chemical and mineralogical composition of clays and metakaolin.

Oxide Composition (mass%)	HC	KC	SC	Phase	SC	KC	HC
SiO_2_ (%)	51–55	53–55	60–62	Quartz (%)	25–30	13–15	9–13
Al_2_O_3_ (%)	26–30	28–32	25–30	Kaolinite (%)	22–25	62–65	
Fe_2_O_3_ (%)	±4	≤1	≤1	Illite/mica (%)	15–18	20–25	8–10
TiO_2_ (%)	≤1	≤1	≤2	Illite/smectite (%)	22–25	≥1	12–14
CaO (%)	≤0.5	≤0.5	≤0.5	Plagioclase (%)		≤1	±12
MgO (%)	≤1	≤0.5	≤0.5	Anatase (%)	≥1	≤1	≤1
K_2_O (%)	≤2	≤2	≤2	Halloysite (%)			55–60
Na_2_O (%)	≤2	≤0.5	≤0.5	kaolinite/smectite (%)	±10		
				D90 (µm)	20	10	18
LOI (%)	±10	±9	±7	BET (m^2^/g)	30.6	24.1	35.9

## Data Availability

The data presented in this study are available in Supplementary Materials.

## Supplementary Materials

The following are available online. Figure S1: Results of Integrity test of samples SC_SA (a), KC_SA (b) and HC_SA (c) after 24H in water, as example of good resistance; Figure S2: Samples obtained with SC clay, KC clay, HC clay and 20% WG, as an example of the good quality of materials; Figure S3: Values of pH of samples made with HC clay (a), SC clay (b) and KC clay (c); Figure S4: DTA and TGA curve of clays as-received of KC (a), SC (b), HC (c); Figure S5: SEM micrograph of (a) SC_NF composition and (b) SC_WG composition after 28 days of curing; Figure S6: SEM micrograph of (a) HC_NF composition and (b) HC_MK composition after 28 days of curing; Table S1: Elemental composition (Atom %) from EDS analysis of KC_NF and KC_NF matrix.

## Sample Availability

Not available.
