# Supplementary material for: Effect of the Introduction of Reactive Fillers and Metakaolin in Waste Clay-Based Materials for Geopolymerization Processes
Source: Molecules. 2021 Mar 2;26(5):1325. doi: 10.3390/molecules26051325 (PMC7958330; doi:10.3390/molecules26051325)
Supplement: Supplementary file 1 [file molecules-26-01325-s001.pdf]

Supplementary Materials

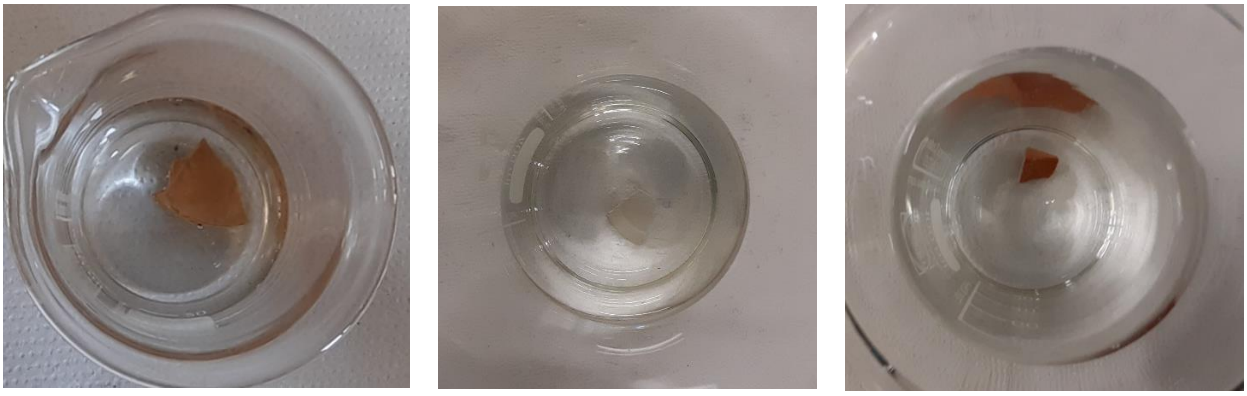

(a) (b) (c)

Figure S1. Results of Integrity test of samples SC_SA (a), KC_SA (b) and HC_SA (c) after 24h in water, as example of good resistance.

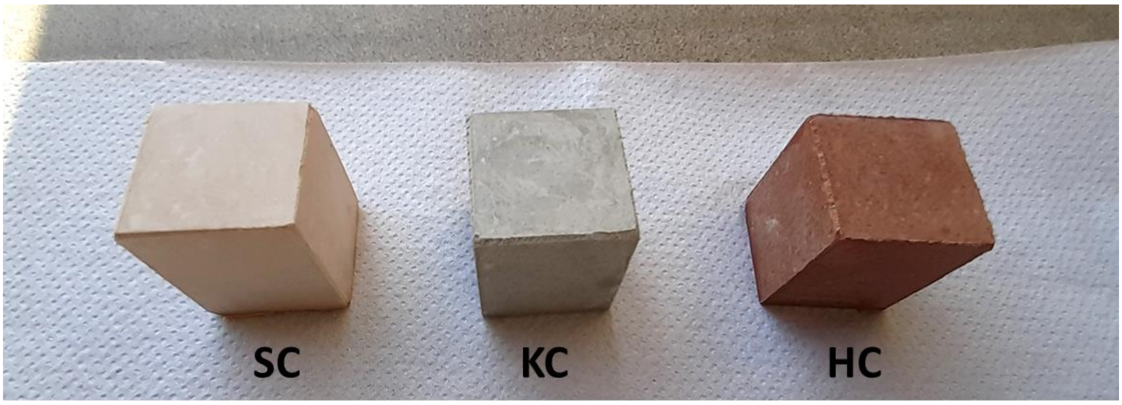

Figure S2. Samples obtained with SC clay, KC clay, HC clay and 20% WG, as an example of the good quality of materials.

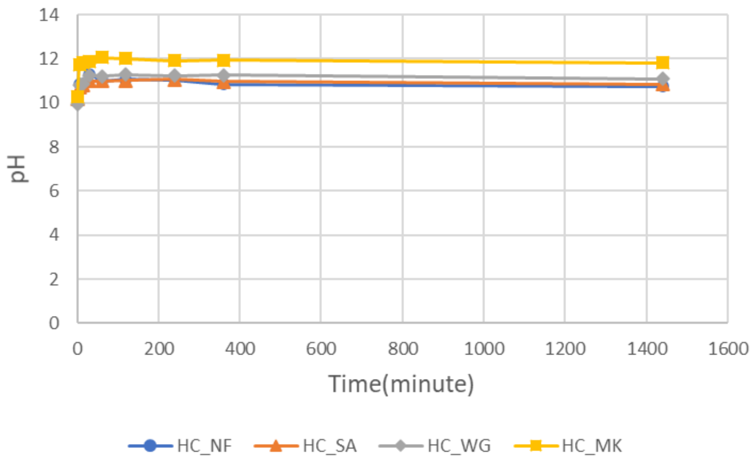

(a)

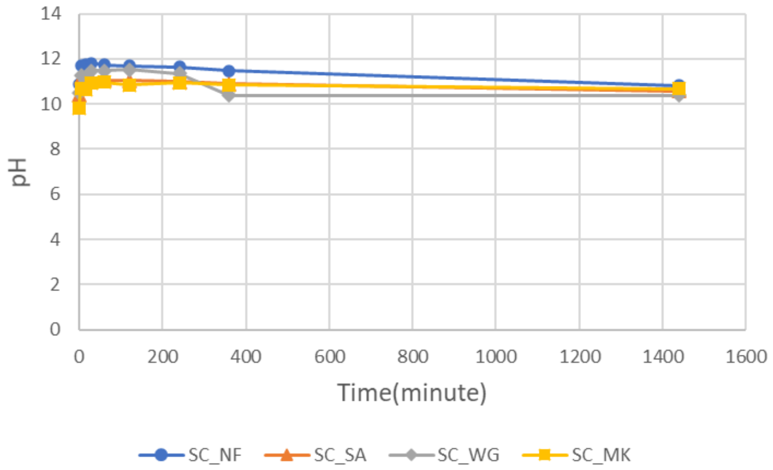

(b)

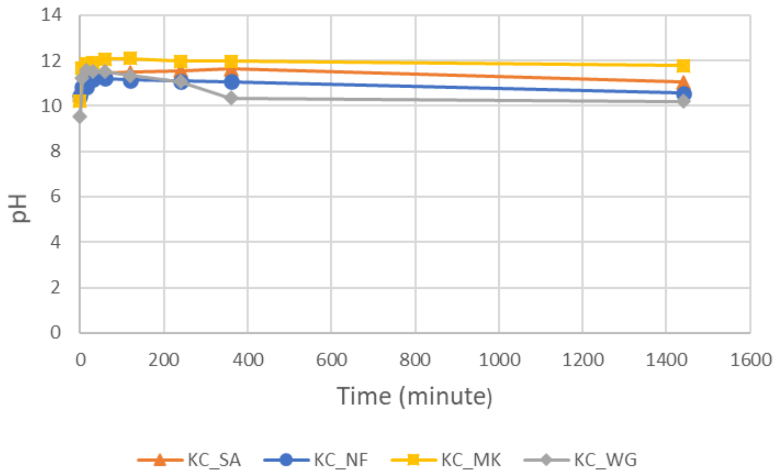

(c)

Figure S3. Values of pH of samples made with HC clay(a),SC clay(b) and KC clay(c)

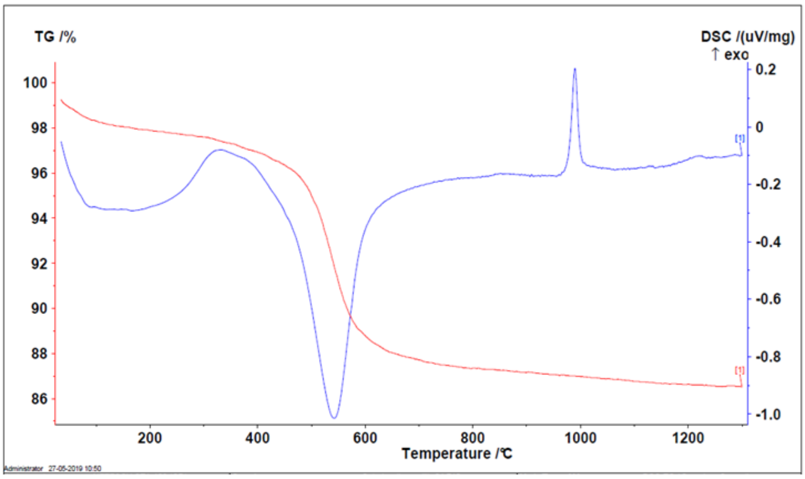

(a)

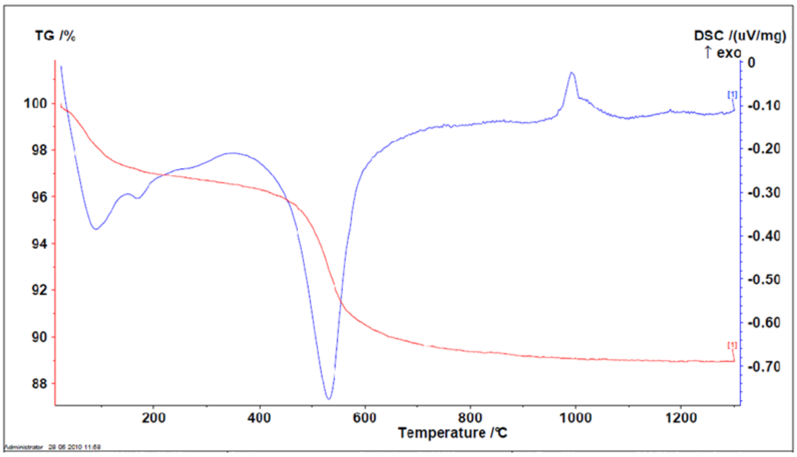

(c)

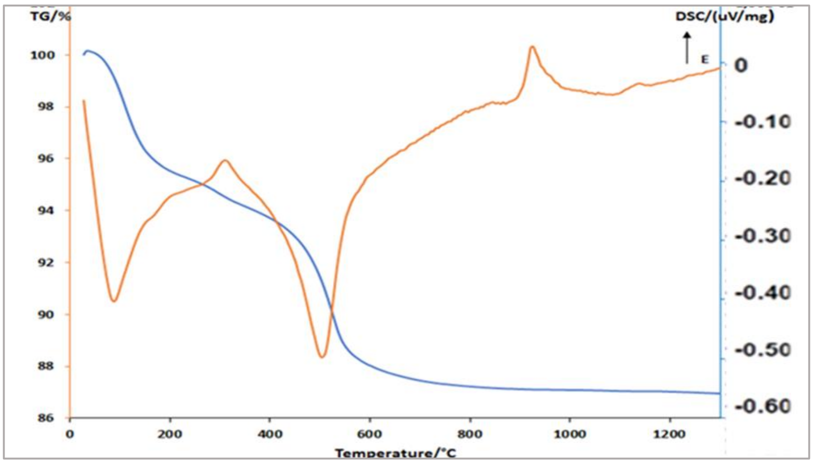

(b)

Figure S4. DSC and TG curves of clays as-received of KC(a), SC(b), HC (c).

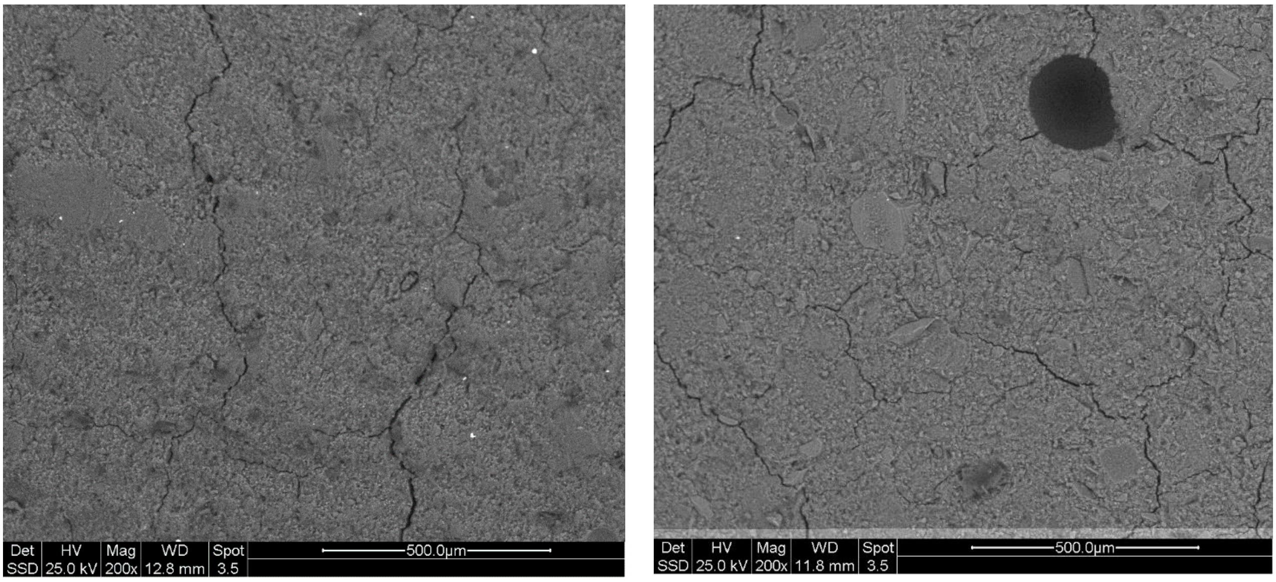

(a) (b)

Figure S5. SEM micrograph of (a) SC_NF composition and (b) SC_WG composition after 28 days of curing

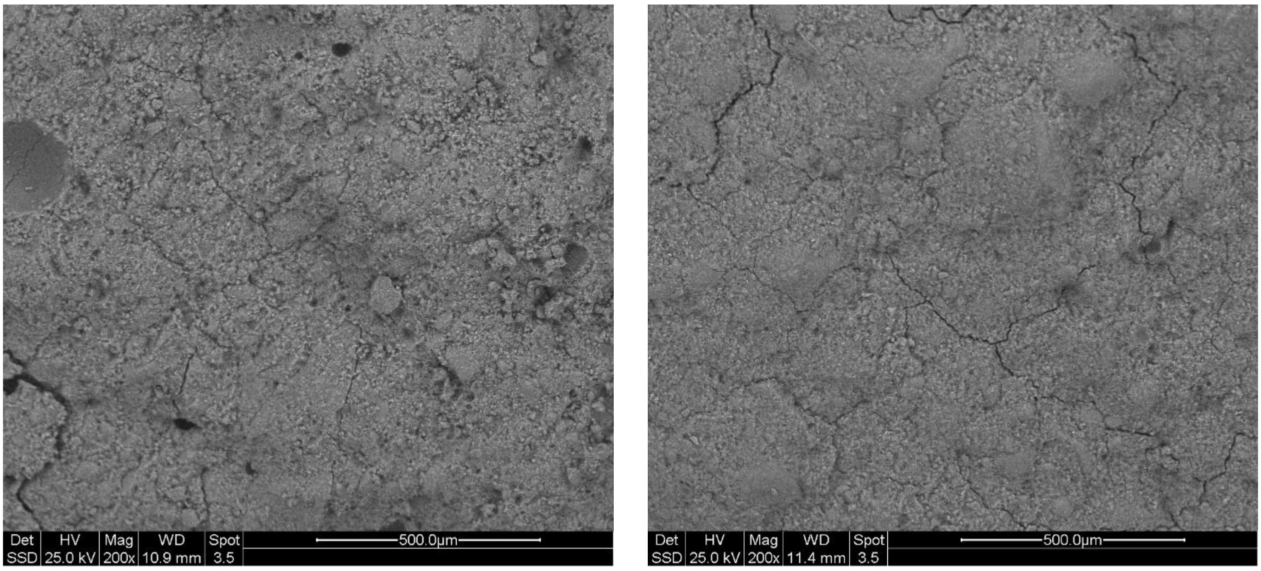

(a) (b)

Figure S6. SEM micrograph of (a) HC_NF composition and (b) HC_MK composition after 28 days of curing

Table S1. Elemental composition (Atom %) from EDS analysis of KC_NF and KC_NF matrix.

KC_NF (atm %)	O	Al	Si	K	Na
	67.01	9.33	16.52	0.5	6.57
	66.18	9.84	17.03	0.64	6.31
	67.05	8.96	17.07	0.77	5.92
	67.57	9.43	16.56	0.66	5.49
Average (%)	66.95	9.39	16.79	0.64	6.07
KC_WG (atm %)	66.86	8.24	16.93	0.43	7.31
	67.23	8.25	16.31	0.55	7.4
	66.74	8.22	16.77	0.66	7.35
	66.71	8.42	16.44	0.58	7.63
Average (%)	66.88	8.28	16.61	0.55	7.42
